# Supplementary material for: Electrical Impedance Spectroscopy as a Tool to Detect the Epithelial to Mesenchymal Transition in Prostate Cancer Cells
Source: Biosensors (Basel). 2024 Oct 15;14(10):503. doi: 10.3390/bios14100503 (PMC11506005; doi:10.3390/bios14100503)
Supplement: Supplementary file 1 [file biosensors-14-00503-s001.zip › biosensors-3157241-supplementary.pdf]

biosensors

Supplementary Materials

Electrical Impedance Spectroscopy as a Tool to Detect the Epithelial to Mesenchymal Transition in Prostate Cancer Cells

Lexi L. C. Simpkins [1,2], Luis A. Henriquez [1,2], Mary Tran [1,2] and Tayloria N. G. Adams [1,2,3,4,*]

[1] Department of Chemical and Biomolecular Engineering, University of California Irvine, Irvine, CA 92697, USA; crowelll@uci.edu (L.L.C.S.); lhenriq1@uci.edu (L.A.H.); phuctt2@uci.edu (M.T.)
[2] Sue and Bill Gross Stem Cell Research Center, University of California Irvine, Irvine, CA 92697, USA
[3] Department of Biomedical Engineering, University of California Irvine, Irvine, CA 92697, USA
[4] Department of Materials and Science Engineering, University of California Irvine, Irvine, CA 92697, USA
* Correspondence: tayloria@uci.edu

The workflow for quantifying the fluorescence intensity of the immunofluorescent stains is illustrated. Images are imported into ImageJ; split into their red, green, and blue channels; and analyzed using only the green channel. ROIs are drawn to measure the fluorescence intensity, and the mean intensity is calculated for more analyses.

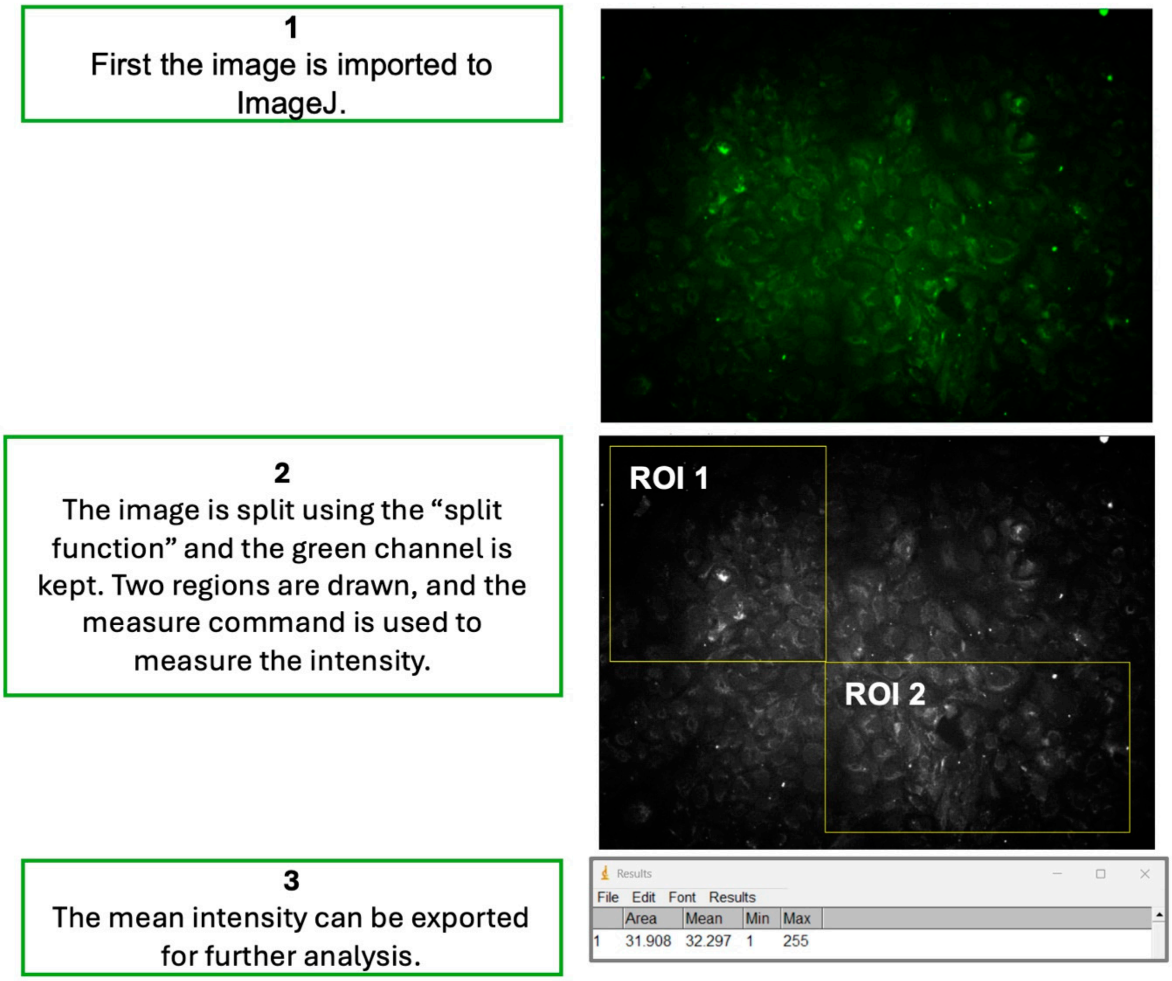

Figure S1. Workflow for quantifying fluorescence intensity of immunofluorescent stains.

EIS and the 3DEP analyzer were used to determine the electrical signature of LNCaP cells without and with EMT treatment. Subtle differences in the electrical signature were observed at specific frequencies, denoted with black dashed line boxes, for both the DEP and impedance spectra.

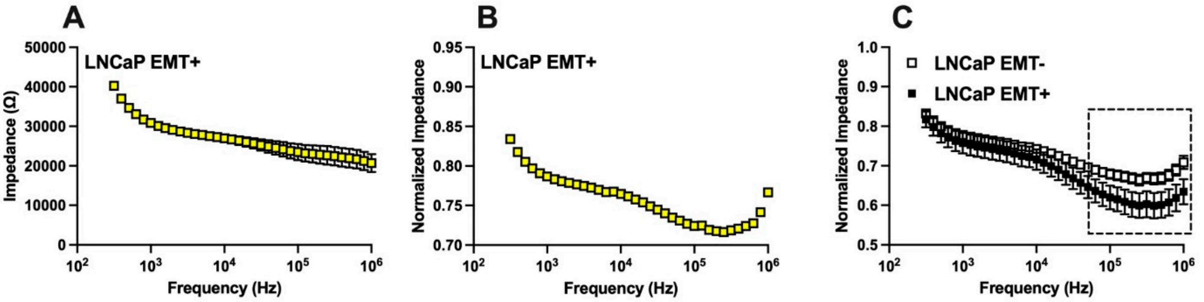

Figure S2. EIS analysis of EMT-treated LNCaP cells. (A) Unnormalized and (B) normalized EIS spectrum of LNCaP EMT+ cells (n=1). (C) Average spectra of LNCaP EMT- cells and LNCaP EMT+ cells.

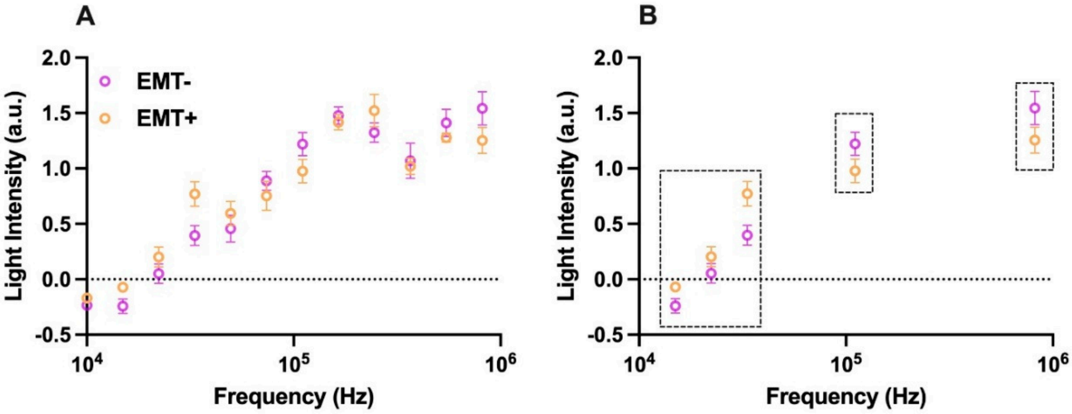

Figure S3. 3DEP analysis of EMT-treated LNCaP cells. (A) Average light intensity response spectra of LNCaP EMT- and EMT+ cells. (B) Black boxes highlight specific frequencies that emphasize differences in electrical signature between EMT- and EMT+ conditions.

Impedance signal ratios were calculated for PC3, DU145, and LNCaP cells to assess the ability of EIS to detect EMT. Deviations from a ratio of one indicate a change in cell state, as guided by the interpretation framework provided. For PC3 and DU145 cells, the ratios of EMT- cells/epithelial control and epithelial control/mesenchymal control provide upper and lower bounds, respectively. More specifically, the EMT- cell/EMT+ cell ratio and the EMT+ cell/mesenchymal control ratio suggest a change in cell state for DU145 and LNCaP cells. In contrast, the EMT- cell/EMT+ cell ratio for PC3 indicates no significant change in the cells following EMT treatment. The epithelial control/mesenchymal control ratio serves as a baseline for interpreting the impedance signal.

Table S1. Impedance signal ratio analysis of EMT-treated PCCs.

Impedance Signal Ratios	PC3[a]	DU145	LNCaP
EMT- cells/epithelial control	1.126	1.011	0.793
EMT- cells/EMT+ cells	1.058	0.950	1.109
EMT+ cells/mesenchymal control	0.966	0.965	0.649
epithelial control/mesenchymal control	0.907	0.907	0.907

Table S2. Interpretation of impedance signal ratios for EMT analysis.

Ratios	Ideal Values	Conclusion
Native (EMT-)/Epithelial Control (EMT-)	Ratio close to 1 (minimal deviation)	Similar impedance suggests no significant change in cell state
Native (EMT-)/Suspected EMT (EMT+)	Ratio > 1 or ratio < 1 (larger deviation)	A deviation from 1 suggests possible EMT occurrence
Suspected EMT (EMT+)/Mesenchymal Control (EMT+)	Ratio close to 1 (minimal deviation)	Similar impedance to mesenchymal control supports EMT
Epithelial Control (EMT-)/Mesenchymal Control (EMT+)	Ratio > 1 or ratio < 1 (larger deviation, provides a baseline difference)	Device performance check based on control properties

The EIS analysis was completed on mixtures of PC3 EMT-/+, DU145 EMT-/+, and LNCaP EMT-/+ cells. Differences are observable at higher frequencies for DU145 and LNCaP cells across EMT-, EMT+, and EMT-/+ conditions. At lower frequencies, there is considerable overlap among these conditions. In the case of PC3 cells, the impedance of the EMT-/+ mixture is similar to those of EMT- cells. The buffer is included for reference.

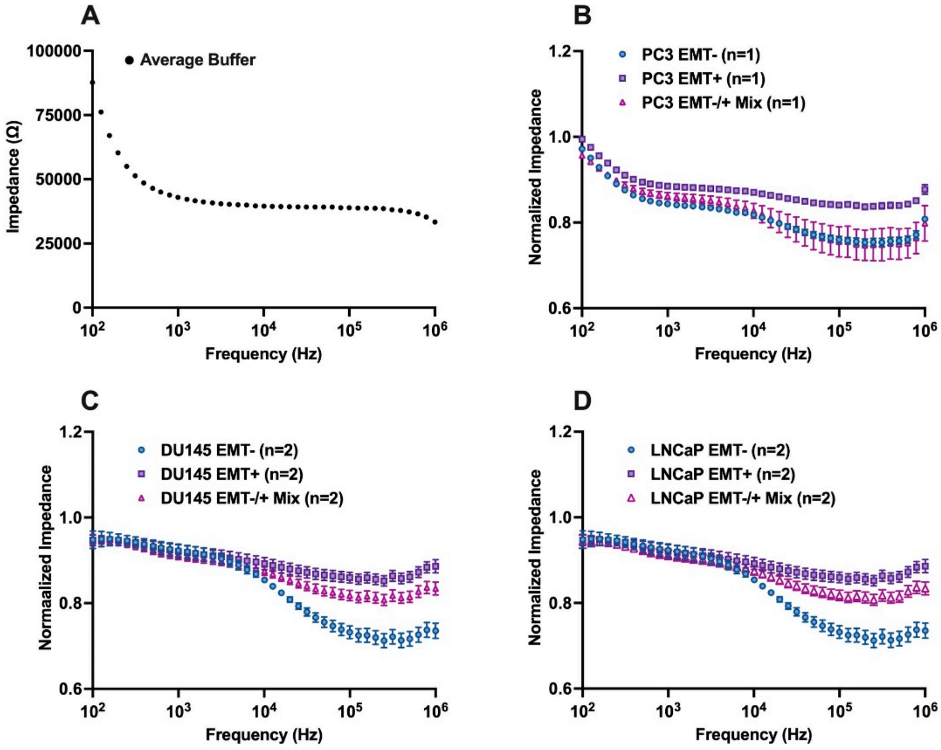

Figure S4. EIS analysis of mixtures of EMT- and EMT+ cells. (A) Impedance spectra of average buffer. Normalized impedance spectra of mixtures for (B) PC3 (n=1), (C) DU145 (n=2), and (D) LNCaP cells (n=2).

A complementary protein expression analysis was conducted on PC3, DU145, and LNCaP cells to assess EMT status. EMT classification as 'true' or 'false' was determined based on E-cadherin and vimentin expression across EMT- and EMT+ states. This analysis supports the fluorescent intensity results in Figure 5, confirming a 'false' EMT status for PC3 cells and a 'true' EMT status for DU145 and LNCaP cells.

Table S3. The image classification of EMT status for cells. A "true" classification requires a ✓ mark in the EMT- E-cadherin box and a ✓ mark in the EMT+ Vimentin box. A "false" classification includes all other combinations. A ✓ mark indicates that most cells in the image are positive for the protein and a × indicates that most cells in the image are negative for the protein.

	Image	EMT- E-cadherin	EMT- Vimentin	EMT+ E-cadherin	EMT+ Vimentin	Classification (True/False for EMT)
PC3	1	✓	✓	✓	×	False
	2	✓	✓	✓	×	False
	3	✓	✓	✓	✓	False
DU145	1	✓	×	×	✓	True
	2	✓	×	×	✓	True
	3	✓	×	×	✓	True
LNCaP	1	✓	×	×	✓	True
	2	✓	✓	✓	✓	False
	3	✓	×	×	✓	True

For the DU145 cells, ZO-1 was used as an additional indicator of EMT. A reduction in ZO-1 protein expression was observed following EMT treatment, indicating that the DU145 cells transitioned from an epithelial phenotype to a mesenchymal phenotype.

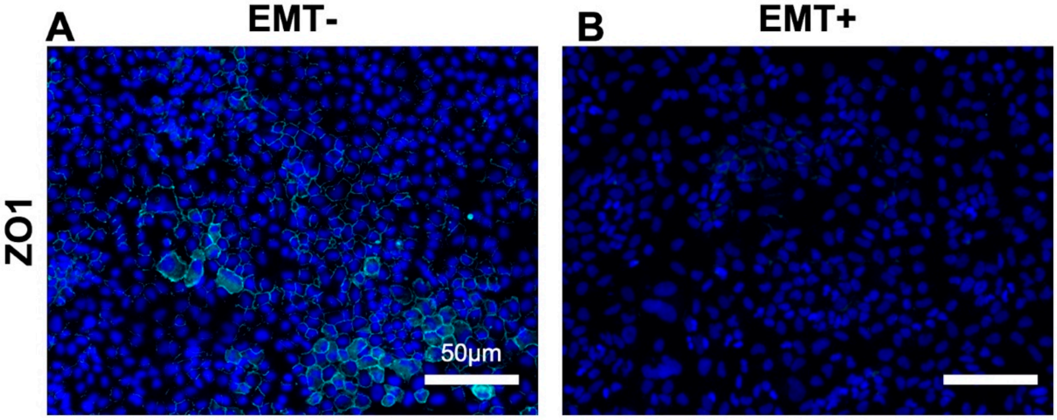

Figure S5. Immunofluorescent staining of DU145 cells without and with EMT treatment (EMT- and EMT+, respectively). The staining highlights the protein expression of the epithelial marker ZO-1.

For the LNCaP cells, N-cadherin served as another indicator of EMT. An increase in N-cadherin protein expression was observed with EMT treatment, suggesting that the LNCaP cells underwent a shift from an epithelial phenotype to a mesenchymal phenotype.

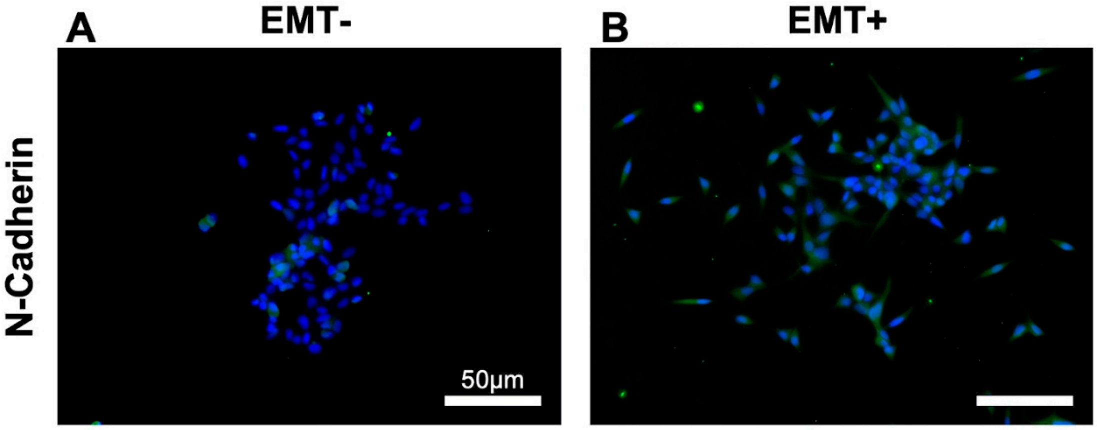

Figure S6. Immunofluorescent staining of LNCaP cells without and with EMT treatment (EMT- and EMT+, respectively). The staining highlights the expression of the mesenchymal marker N-cadherin.

Guidance for EMT Detection: This study focuses on EMT detection using EIS; below, we provide general EMT detection procedures. Follow our Materials and Methods to generate EIS data and select the appropriate controls for comparison. To facilitate cross-comparisons of examined cells, normalize the impedance to the buffer (refer to Equation 1).

Key early indicators of EMT are characterized by differences in the normalized impedance spectra, specifically (a) the distinction between the spectra of cells suspected of undergoing EMT and their native cell state, and (b) the distinction between the spectra of the cells suspected of undergoing EMT and mesenchymal control cells. Confirm statistical significance in the impedance spectra by averaging the normalized impedance over the entire tested frequency range, or a narrower range without spectral overlap, for each cell condition assessed, cells suspected of undergoing EMT, the native cell state, the mesenchymal control, and the epithelial control, for completeness. Proceed with cell populations that show statistically significant differences compared to controls (native, mesenchymal, and epithelial cells) and compute impedance signal ratios. These ratios include native state cells divided by epithelial controls, native state cells divided by cells suspected of undergoing EMT, cells suspected of undergoing EMT divided by mesenchymal controls, and epithelial controls divided by the mesenchymal controls. For this study, statistical significance in the impedance signal is assessed in Figures 3E and 3F with the ratio comparisons presented in Supplemental Table S1 above.

Key later indicators of EMT include assessing cell morphology and protein expression. Cells should be examined in their native state and after suspected EMT for changes in the shape and expression of hallmark EMT proteins, such as E-cadherin, N-cadherin, vimentin, and ZO-1. At a minimum, a statistical analysis should be performed on the protein expression data. A 'true/false' framework can be used to classify images based on protein expression, where a ‘true’ classification for undergoing EMT requires cells to be positive for E-cadherin in the EMT- state and positive for vimentin in the EMT+ state. Conversely, images labeled as ‘false’ for EMT will include all other combinations of epithelial and mesenchymal marker expression across EMT- and EMT+ states. For this study, the morphology assessment appears in Fig. 4 and some representative ‘true/false’ classifications have been made in Supplemental Table S3 above to complement the RGB analysis in Fig. 5.

Synthesizing the results of both early and later indicators (impedance assays vs. phenotypic assays) provides a comprehensive assessment of whether cells have undergone EMT. Although this EMT detection procedure combines impedance assays with phenotypic assays, the goal is to reliably establish the electrical signature of cells undergoing EMT, allowing phenotypic assays to serve as a complementary technique that can be selectively used rather than a required step.

This detection procedure is applicable to the cells studied here and is potentially translatable to other cancer types or other cells undergoing EMT.
